# Bioreactors for lignocellulose conversion into fermentable sugars for production of high added value products

**DOI:** 10.1007/s00253-015-7125-9

**Published:** 2015-11-16

**Authors:** Rossana Liguori, Valeria Ventorino, Olimpia Pepe, Vincenza Faraco

**Affiliations:** Department of Chemical Sciences, University of Naples Federico II, Complesso Universitario Monte S. Angelo via Cintia 4, 80126 Naples, Italy; Department of Agriculture, University of Naples Federico II, Portici, Italy

**Keywords:** Single and double helical ribbon impeller, Rushton impeller, S-shaped impeller, Anchor impeller, Pitched-blade impeller, Peg-mixer, Paddle blade magnetic impeller

## Abstract

Lignocellulosic biomasses derived from dedicated crops and agro-industrial residual materials are promising renewable resources for the production of fuels and other added value bioproducts. Due to the tolerance to a wide range of environments, the dedicated crops can be cultivated on marginal lands, avoiding conflict with food production and having beneficial effects on the environment. Besides, the agro-industrial residual materials represent an abundant, available, and cheap source of bioproducts that completely cut out the economical and environmental issues related to the cultivation of energy crops. Different processing steps like pretreatment, hydrolysis and microbial fermentation are needed to convert biomass into added value bioproducts. The reactor configuration, the operative conditions, and the operation mode of the conversion processes are crucial parameters for a high yield and productivity of the biomass bioconversion process. This review summarizes the last progresses in the bioreactor field, with main attention on the new configurations and the agitation systems, for conversion of dedicated energy crops (*Arundo donax*) and residual materials (corn stover, wheat straw, mesquite wood, agave bagasse, fruit and citrus peel wastes, sunflower seed hull, switchgrass, poplar sawdust, cogon grass, sugarcane bagasse, sunflower seed hull, and poplar wood) into sugars and ethanol. The main novelty of this review is its focus on reactor components and properties.

## Introduction

The use of crops as renewable sources of energy and compounds in alternative to fossil resources can promote a sustainable development avoiding the problems of shortage of fossil feedstock (Liguori et al. 2013; Kajaste 2014), but it generates problems in the agricultural market since their cultivation increases the lands subtracted to the food production and rising global food prices (Scheidel and Sorman 2012).

To limit the competition between the food- and the non food-crop lands, the cultivation of dedicated energy crops in marginal lands non appropriate for the traditional food crops is spreading throughout the world (Popp et al. 2014). It is noteworthy that the large-scale cultivation of dedicated crops, such as the perennial biomass *Arundo donax*, have favorable effects on the environment, since it improves soil fertility and reduces soil erosion (Fagnano et al. 2015). Moreover, lignocellulosic agro-industrial residual materials represent a further alternative of cheap sources to further minimize the conflict of food versus fuel. They avoid the displacement of food crops and the issues related to the deforestation, limiting the negative impacts on the environment (Iqbal et al. 2013).

**Fig. 1:**
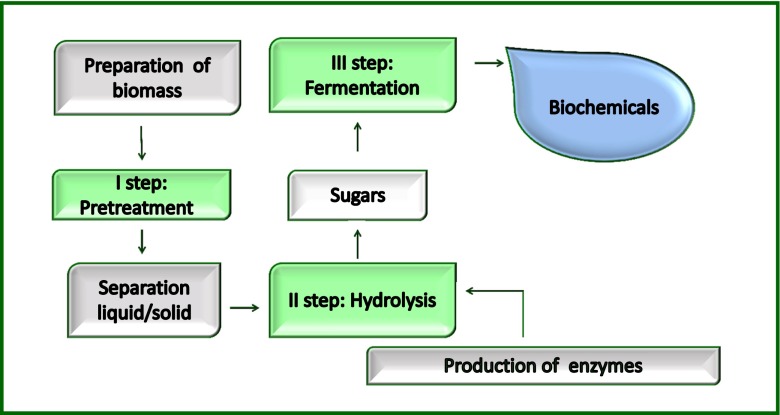
Main steps of process of lignocellulosic biomass conversion

Due to the high cellulose and hemicellulose contents (an average of 40 and 30 %, respectively) (Limayem and Ricke 2012), the dedicated crops and the residual materials can be converted in different value-added products, such as fermentable sugars (Mezule et al. 2015) and bioethanol or other bioproducts obtained by sugars fermentation (Shahsavarani et al. 2013). The complexity of the lignocellulosic macromolecular structure requires a bioconversion process consisting of three phases (Fig. 1). The first step is the biomass pretreatment needed to remove the lignin and make the polysaccharides more accessible to the further hydrolysis and it is considered generating the most negative impact on the environment, due to the high energetic inputs. The polysaccharides are then subjected to hydrolysis into monosaccharides mainly performed by hydrolytic enzymes during the second step, which is the most costly step of the overall process due to the high costs of the enzymes. In the last fermentation step, the fermentable sugars are converted into the targeted added value bioproducts (Jørgensen et al. 2007a).

Several efforts are under way to improve both the molecular systems, hydrolytic enzymes (Amore et al. 2012, 2013, 2015; Salmon et al. 2014; Weingartner Montibeller et al. 2014; Giacobbe et al. 2014), and microorganisms (Liguori et al. 2015; Ventorino et al. 2015), and bioreactor systems adopted for the biomasses bioconversion process in order to solve the environmental and economical issues of the process (Wang et al. 2011; Khoo 2015).

**Fig. 2:**
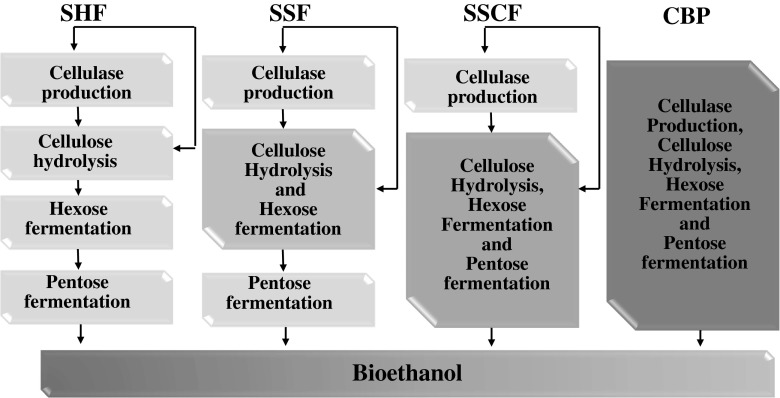
Processes of sugars and bioethanol production. SHF separate hydrolysis and fermentation, SSF simultaneous hydrolysis and fermentation, SSCF simultaneous saccharification and co-fermentation of both hexoses and pentoses

This review summarizes the last advances in the bioreactor field, with main focus on the new configurations and the agitation systems, for conversion of dedicated energy crops and residual materials into sugars and ethanol by separate hydrolysis and fermentation (SHF), simultaneous saccharification and fermentation (SSF), simultaneous saccharification and co-fermentation (SSCF) and consolidated bioprocessing (CBP) (Fig. 2).

The attention was focused on the dedicated crops *Arundo donax* (Table 1), corn stover (Table 2), wheat straw (Table 3) and other residual materials (Table 4). The main novelty of this review is its focus on reactor components and properties.

**Table 1:** Main characteristics of some examples of reactors for conversion of *Arundo donax*

Biomass	Reactor system	Agitation	Tank volume	Configuration process	Glucose concentration/yield/productivity	Reference
*Arundo donax*	Stirred tank bioreactor	Anchor impeller	3.0 L	Enzymatic hydrolysis	∼40 % of glucose yield at constant impeller speed	Palmqvist and Lidén 2012
*Arundo donax*	–	Blade impeller with three blades at an angle of 45 °	–	Enzymatic hydrolysis	∼60 % of glucose yield	Kadić et al. 2014

**Table 2:** Main characteristics of some examples of reactors for conversion of corn stover

Reactor system	Agitation	Tank volume	Configuration process	Glucose concentration/yield/productivity	Reference
Corn stover					
Horizontal rotating bioreactor (HRR) and vertical stirred-tank reactor (VSTR)	Mixing blade in the HRR system and a double helical ribbon impeller in the VSTR system	–	Batch and fed-batch	86 g glucose kg^−1^ of dry matter in the HRR through the batch hydrolysis and 73 g glucose kg^−1^ of dry matter in the batch VSTR system through the batch hydrolysis	Du et al. 2014
8-L scraped surface bioreactor (SSBR)	3 scraping blades	–	Enzymatic hydrolysis	Glucose yield of 60 %	Dasari et al. 2009
Stirred-tank reactor	–	–	Enzymatic hydrolysis	50 g glucose kg^−1^ of cellulose	Kadam et al. 2004
5 bioreactors connected in series for the CSTR process	–	–	SHF and SSCF in batch and continuous mode	Volumetric productivity of 0.25 g L^−1^ h^−1^ and 0.20 g L^−1^ h^−1^ in batch SSCF and SHF, respectively. Maximum volumetric productivity of 0.46 g L^−1^ h^−1^ in continuous SSCF by using a CSTR	Jin et al. 2013
–	Double helical impeller and Rushton impeller	5.0 L	SSF	51.0 g L^−1^ of ethanol in the SSF by using the double helical impeller and 43.9 g L^−1^ in the SSF by using the Rushton impeller	Zhang et al. 2010
	Single helical ribbon impeller		SSF	56.2 g L^−1^ of ethanol	He et al. 2014
Corn slurry					
–	–	3.0 L	Batch cSSF and the 3-stage CSTR cSSF	Ethanol yield of 70 % in the batch and in the 3-stage CSTR compared to the 0.18 g L^−1^ h^−1^ batch one. Maximum productivity of 0.4 g L^−1^ h in the single-vessel CSTR	Brethauer et al. 2014
Ultrasonic reactor equipped with a donut-shaped horn	–	–	Batch and continuous flow	61.6 g L^−1^ in batch system and 30.2 g L^−1^ in continuous flow	Montalbo-Lomboy et al. 2010b

**Table 3:** Main characteristics of some examples of reactors for conversion of wheat straw

Reactor system	Agitation	Tank volume	Configuration process	Glucose^a^ or ethanol^b^ concentration/yield/productivity	Reference
Stirred tank reactor	Segmented helical stirrer	–	Enzymatic hydrolysis	^a^Glucose yield of 76 % and 110 g glucose kg^−1^ biomass	Ludwig et al. 2014
48 parallel stirred tank bioreactors	S-shaped impellers	10 mL	Enzymatic hydrolysis	^a^∼111 mg glucose g^-1^ dry matter	Riedlberger and Weuster-Botz 2012
–	System of rotating paddle	5.0 L	Batch process, Sequential hydrolysis and solids-recycling processes	^**a**^Glucose yield of 59 % in batch process, 53 % in sequential hydrolysis, and 63 % in solids-recycling processes. Productivity of 54 and 30 % higher than that reached in the batch system (0.8 g L^−1^ h^−1^) were observed in the sequential hydrolysis and solids-recycling systems, respectively	Pihlajaniemi et al. 2014
Horizontally placed drum divided into 5 independent sections	Three paddlers	–	8 h of liquefaction and pre-saccharification following by 84 SSF	^b^48 g ethanol kg^-1^ of biomass	Jørgensen et al. 2007b
–	–	2.5 L	Fed-batch SSCF and the enzyme feeding SSCF	^b^0.35 g g^−1^ of ethanol yield and 38 g L^−1^ of the ethanol concentration	Olofsson et al. 2010a

**Table 4:** Main characteristics of some examples of reactors for conversion of different biomass residues

Biomass	Reactor system	Agitation	Tank volume	Configuration process	Ethanol concentration (g L^−1^)-yield (%)-Productivity (g L^−1^ h^−1^)	Reference
*Prosopis juliflora* (Mesquite)	Stirred tank bioreactor	Rushton impeller	3.0 L	Batch SHF	34.78 g L^−1^ - 0.45 g g^−1^ - 3.16 g L^−1^ h^−1^	Gupta et al. 2012
*Prosopis juliflora* (Mesquite)	Stirred tank bioreactor	Rushton impeller	3.0 L	Fed-batch SHF	52.83 g L^−1^ - 0.45 g g^−1^ - 4.40 g L^−1^ h^−1^	Gupta et al. 2012
Agave bagasse	Mini-bioreactor	Peg-mixer	6 × 30 mL	SHF	64 g L^−1^	Caspeta et al. 2014
Citrus peel waste	Limonene removal column-immobilized cell reactor system	–	80 mL	SHF	from 14.4 to 29.5 g L^−1^ (ethanol yields 90.2–93.1 %)	Choi et al. 2015
Sunflower seed hull	Batch culture bioreactor system	Teflon-glass impeller/paddle blade magnetic impeller	0.6 L	SHF	9.66 g L^−1^ and ethanol yield 0.41 g g^−1^	Okur and Saraçoglu 2006
Switchgrass	Steam-jacketed fermenter	Rushton impeller	50 L	Batch SFF	73 %	Isci et al. 2009
Switchgrass	Steam-jacketed fermenter	Blade axial flow impeller	350 L	Fed-batch SFF	74 %	Isci et al. 2009
*Miscanthus*	Twin screw reactor-fermenter	Rushton impeller (attached at the cap)	5 L	Fed-batch SFF	74.5 g L^−1^ - 89.5 % - 1.4 g L^−1^ h^−1^	Han et al. 2014
Poplar sawdust	Continuous twin screw reactor-fermenter	–	4 × 1 L	Fed-batch SFF	39.9 g L^−1^	Kim et al. 2013
Cogon grass	Rotary drum reactor	rotation	5 L	SSF	19.1 g^−1^ L^−1^ (yield 76.2 %)	Lin and Lee 2011
Sugarcane bagasse	Rotary drum reactor	rotation	100 L	SSF	24.6 g L^−1^ (yield 79 %)	Lin et al. 2013
Spruce chips	Three-unit integration system (hydrolysis reactor, filtration/pump system, fermentation reactor)	helical stirrer	2.5-L hydrolysis reactor/1.5 L fermentation reactor	SSFF	31.1 g L^−1^ ethanol, corresponding to 85.0 % theoretical yield	Ishola et al. 2013
Poplar wood	Stirred vessel fermentor (Biostat, Sartorius)	Rushton-type stirrer blades	2 L	CBP	34.8 mM (1.06 g L^−1^)	Svetlitchnyi et al. 2013

### Bioreactors for *Arundo donax* conversion

#### Production of sugars from SHF

**Fig. 3:**
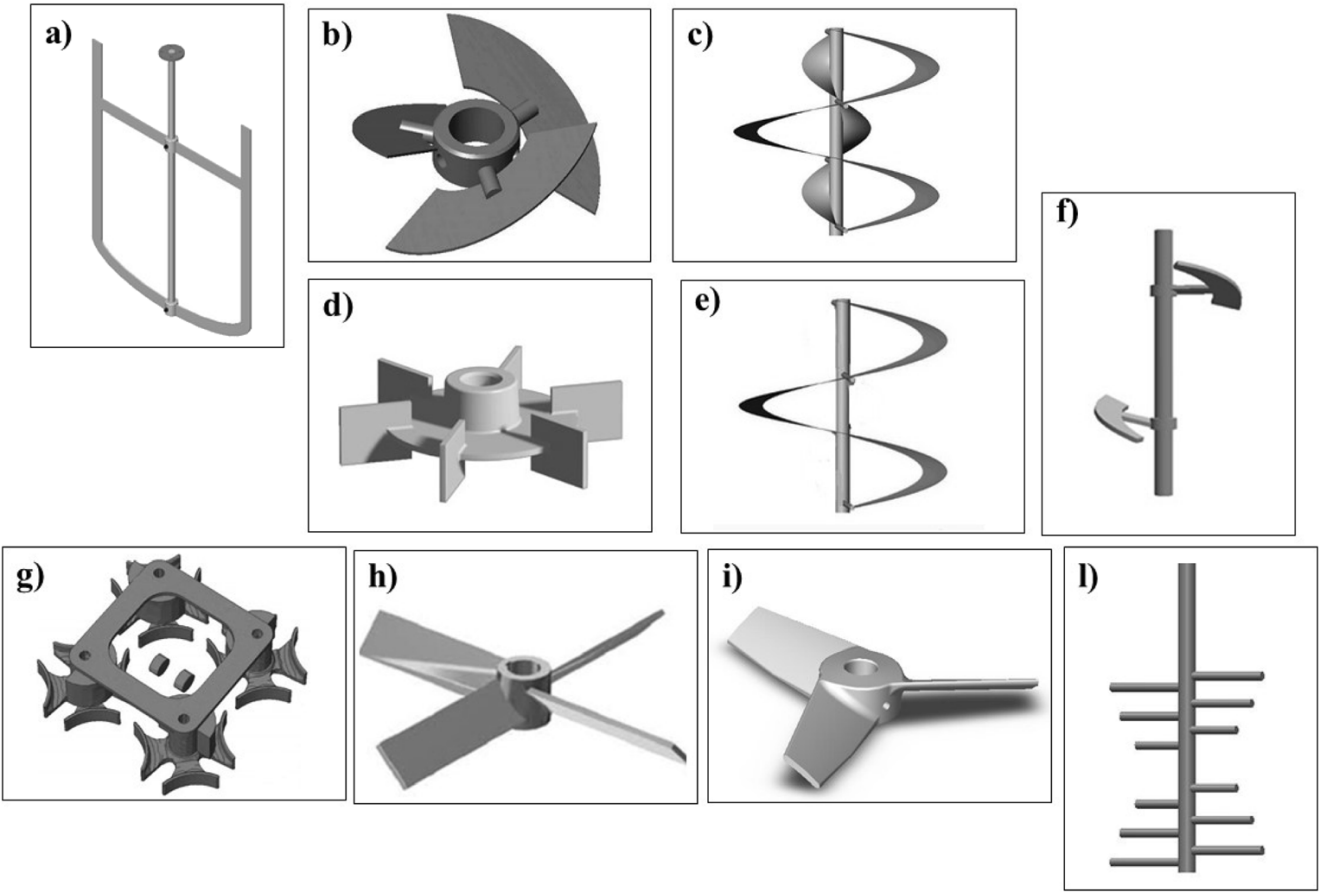
Agitation systems used in the enzymatic hydrolysis and fermentation of lignocellulosic biomasses. **a)** Anchor impeller. **b)** Blade impeller with three blades at an angle of 45 °. **c)** Double helical ribbon impeller. **d)** Rushton impeller. **e)** Single helical ribbon impeller. **f)** Segmented helical stirrer. **g)** S-shaped impellers. **h)** Rotating paddle. **i)** Three paddlers. **l)** peg mixer

The energy crop *Arundo donax* was investigated by Palmqvist and Lidén (2012), in comparison with spruce, to evaluate the influence of water-insoluble solids (WIS) content on glucose yield during the hydrolysis. Both biomasses were steam pretreated and tested at WIS content of 10, 15, and 20 %. The process was performed in a 3-L stirred tank bioreactor (Belach Bioteknik, Stockholm, Sweden), supplied with an **anchor impeller** (Fig. 3a) (13-cm diameter and 2-cm blade width). The Cellic CTec2 (Novozymes, Denmark) (0.1 g solution g^−1^ WIS) was adopted as enzyme preparation. They tested two different methods, the first keeping constant the impeller speed at 10 rpm, and the second one keeping constant the impeller power input, in order to identify the best system to be applied for the hydrolysis of biomasses at high WIS concentration. They demonstrated that, at a fixed impeller speed of 10 rpm, the WIS content did not influence the energy input for *Arundo donax*, while a higher overall energy input was required for the hydrolysis of the spruce because, for the latter system, a strong correlation between initial WIS content and energy input was observed. This is explained by a quick drop in torque and viscosity that occurred during the saccharification of *Arundo donax*, as reported for other biomasses (Dasari et al. 2009), and that was much less noticeable for the spruce. In detail, when the impeller speed was kept constant and the WIS concentration was increased from 10 to 20 %, the glucose yield decreased from ∼40 to ∼27 % for *Arundo donax* and increased from ∼20 to ∼30 % for the spruce. Otherwise, when the impeller power was kept constant, the glucose yield was equivalent to that obtained at constant impeller speed for *Arundo donax*, while an opposite trend was observed for the spruce, since the glucose yield decreased from ∼45 to ∼30 % when the WIS content increase from 10 to 20 %. It could be due to a different shear force in the reactor between the two biomasses. Furthermore, Kadić et al. (2014) investigated the effect of agitation rate on the particle-size distribution (PSD) and glucan release during hydrolysis of the steam pretreated *Arundo donax* and spruce. The 2.5-L Biostat A and Biostat A Plus bioreactors (B. Braun Biotech International, Germany), equipped with a **pitched-blade impeller with three blades at an angle of 45 °** (diameter of 70 mm and a blade width of 20 mm) (Fig. 3b), were used for the hydrolysis. Three impeller speeds 100, 300, and 600 rpm were tested, evaluating their different effects on the biomass-particles mixing. In the case of spruce, the effects of agitation rate were only observed using high WIS content (13 %) at higher speed. In fact, no effects during the hydrolysis were observed at 13 % of WIS at 100 rpm, but when the impeller speed was enhanced up to 600 rpm, an increase in the hydrolysis rate from 20 to 37 % after 48 h took place. This could be explained as a result of strong reduction of particle size that improves the sugars released, increasing the hydrolysable surface area. Otherwise, for *Arundo donax* hydrolysis, a smaller particle size than spruce was observed both at low and high impeller speed; in spite of this, only a slight temporary effect (from 43 % at 100 rpm to 53 % at 600 rpm after 48 h) on the hydrolysis rate at high WIS content (13 %) occurred. After 96 h of hydrolysis, the same rate of ∼60 % was reached in both systems. To investigate if the reduction of particle size was caused by the enzymes action or by the agitation rate, further experiments in which the lignocellulose biomasses at 13 % of WIS were agitated at high revolutions per minute without the enzymes addition were performed by Kadić et al. (2014). For the spruce, the effects of agitation rate on the particle size was strongly evident; in contrast, the reduction of particle size of *Arundo donax* was more influenced by the enzyme’s action than the agitation rate, since the highest size reduction was only observed when the enzymes were loaded. Based on these results, it is important to choose the agitation speed based on the macromolecular structure of the lignocellulosic biomasses and the initial solids loading.

### Bioreactors for corn stover conversion

#### Production of sugars from SHF in batch operation mode

The effect over the yield of sugars obtained during the hydrolysis of the sulfuric acid/steam pretreated corn stover employing two different reactor systems, the horizontal rotating bioreactor (HRR) and the vertical stirred-tank reactor (VSTR), was investigated by Du et al. (2014). The saccharification was performed by using the enzyme Cellic CTec2 from Novozymes, at loading of seven filter paper activity units per gram of dry matter (FPU g^−1^) at 50 °C in both reactor systems, equipped with thermostatic water bath. The material blending was assured by a mixing blade, at maximum constant rotation speed (100 rpm) in the HRR system and by a **double helical ribbon impeller** (Fig. 3c), at variable speed in the VSTR system. During the hydrolysis, a more rapid viscosity reduction occurred in the HRR, allowing better cellulose degrading in comparison with the VSTR. Comparing the batch and fed-batch (adding biomass or biomass/enzymes) enzymatic hydrolysis, it was demonstrated that the best result of 86 g glucose kg^−1^ of dry matter was obtained after 87 h of incubation in the HRR through the batch hydrolysis in comparison with the value of 73 g glucose kg^−1^ of dry matter shown by the batch VSTR system.

It is well known that the use of ultrasound for lignocellulose treatment improves the enzymatic hydrolysis yield (Khanal et al. 2007; Nitayavardhana et al. 2008; Montalbo-Lomboy et al. 2010a). Recently, Montalbo-Lomboy et al. (2010b) investigated the influence of the batch system over the saccharification sugars yield and the particles size of the corn slurry. The STARGEN^TM^ 001 from Genencor, at loading of 456 granular starch hydrolyzing units per gram of substrate (GSHU g^−1^), was added after sonication and was used as enzyme in the hydrolysis step for 3 h in a rotary shaker. The results showed a yield of reducing sugars obtained with the batch system equal to 1.6 g L^−1^.

The high solid processing of corn stover (PCS) represents one of the main drawbacks in the saccharification step. Even though an initial high PCS allows to obtain different advantages, like the reduction of reactor size, it is generally avoided due to the high viscosity shown and the high power required to mix homogenously the substrates. Dasari et al. (2009) designed a 8-L scraped surface bioreactor (SSBR), equipped with three scraping blades, to improve the saccharification of the corn stover at high initial PCS. They compared the glucose liberated in the process by using the bioreactor against the results obtained in 250-mL Erlenmeyer flasks. In this work, the saccharification was performed at 50 °C with 15 FPU of Spezyme CP cellulase enzyme (Genencor) per gram of cellulose, at speed of 250 rpm in flasks and 2 rpm in bioreactor. The latter system allowed to reach a glucose yield of 60 %, a value 10 % higher than that obtained in flasks (50 %) and, within the first hours of the hydrolysis reaction, a quick decrease in torque was observed, allowing a low-energy consumption. This was due to the random radial and angular mixing of the biomass and the homogeneous temperature generated by the horizontal rotation and the scraping of the blades.

It is well known that the enzymatic hydrolysis represents the limiting step of the overall costs of lignocellulose bioconversion process. Thus, several kinetic models to reduce both costs and efforts of the step were generated (Philippidis and Hatzis 1997; Gusakov et al. 1985; Sadana 1992; Kadam et al. 2004. One of them was elaborated and validated considering not only the reaction for conversion of cellulose into glucose and cellobiose, but also for the conversion of the cellobiose into glucose (Kadam et al. 2004). Moreover, parameters like enzyme adsorption, substrate reactivity, temperature, and sugar inhibition were taken into account. Following the model, the sulfuric acid-treated corn stover was saccharified with 45 mg protein per gram of cellulose (CPN commercial cellulase, Iogen Corp., Ottawa, Canada) at 45 °C in 250-mL baffled Erlenmeyer flasks stirred at 130 rpm, or in stirred-tank reactor with impeller speed of 250 rpm. The results demonstrated that the model fitted well to predict the glucose yield both in flasks and in tank reactor. The maximum glucose concentration of around 50 g kg^−1^ of cellulose, after 168 h, was obtained for both systems used. Although the temperature effect was not completely defined by the model, this could be exploited to optimize the saccharification process in silico.

#### Production of sugars from SHF in fed-batch/continuous operation mode

Montalbo-Lomboy et al. (2010b) investigated also the influence of the continuous-flow ultrasonic system over the saccharification sugars yield and the particle size of the corn slurry. Enzyme hydrolysis was performed in an ultrasonic reactor fitted with a donut-shaped horn using the same enzyme amount and process parameters described earlier. In addition, in this system, the corn slurry was localized in the center of the donut-shaped horn where the cavitation was more intense, increasing the liquefaction of the biomass. The results showed an increase up to 2–3 times of the sugars yield in the sonicated samples in comparison with the control and the yield of reducing sugars obtained was 30.2 g L^−1^.

#### Production of ethanol from SSF/CBP in batch operation mode

Brethauer et al. (2014) performed a simultaneous saccharification and fermentation (SSF) of acid-pretreated corn stover in batch system. Five filter paper activity units per gram of glucan of Spezyme CP cellulase (Genencor) and 7.5 CBU g^−1^ of glucan of Novozyme 188 ß-glucosidase (Novozyme) were employed for the saccharification step, while the strain *Saccharomyces cerevisiae* D5A was used for the fermentation at 38 °C. The corn slurry, at low solids concentration, was pumped from the reservoir, equipped with a magnetic stir plate, to the 3-L fermentor together with air bubbles to avoid the accumulation of solid in the tube. They observed an ethanol yield of 70 % and an ethanol productivity of 0.18 g L^−1^ h^−1^.

Jin et al. (2013) evaluated the conversion of ammonia fiber expansion (AFEX)-pretreated corn stover into ethanol by using two different systems: the SHF and the SSCF. An enzymatic mix containing Accellerase 1500, Accellerase XY, and Multifect pectinase (Genencor) at loadings of 24, 6, and 6 mg g^−1^ of glucan, respectively, was used for the hydrolysis of corn stover. A genetically modified strain of *S. cerevisiae* 424A fermenting xylose was employed for the ethanol fermentation. Firstly, they noted that, although more sugars were released in the SHF and the same ethanol yield was reached in both systems (80 and 47 % of glucose and xylose conversion into ethanol, respectively), the volumetric productivity was 0.25 g L^−1^h^−1^ for the batch SSCF compared to the 0.20 g L^−1^h^−1^ of the SHF.

Zhang et al. (2010) designed a reactor with a new agitation system namely double helical impeller (Fig. 3c), in substitution of the common **Rushton impeller** (Fig. 3d), to perform the simultaneous saccharification and fermentation (SSF) of corn stover using high solids loading. Accellerase 1000 from Genencor International (Rochester, NY) at different dosages, and the thermo- and inhibitor-tolerant baker’s yeast mutant *S. cerevisiae* DQ1 were used for the saccharification and fermentation steps, respectively. The experiments were conducted in the 5-L bioreactor, performing a prehydrolysis step for 12 h at 50 °C followed by the SSF step at 37 °C to allow the *S. cerevisiae* DQ1 growth. They demonstrated how the new agitation system improved the ethanol yield: 51.0 g L^−1^ of ethanol were obtained at the end of SSF by using the double helical impeller, respect to the 43.9 g L^−1^ reached in the reactor equipped with the Rushton impeller. The best yield was obtained using the double helical impeller due to the better mixing, and this system also reduced the overall process energy demand. Moreover, they reported that at 30 % of solids loading, the ethanol concentration reached 40.0, 59.3, and 64.6 g L^−1^ at enzyme dosages of 7.0, 15.0, and 30.0 FPU g^−1^ of dry matter, respectively. Recently, He et al. (2014) performed the dry acid pretreatment of corn stover at high solids concentration by using a reactor equipped with a **single helical ribbon impeller** (Fig. 3e), demonstrating as the steady helically agitation leads to increase sugars and ethanol yields production. The corn stover was treated with 2.5 % H_2_SO_4_ for 3 min at 185 °C in agitation, followed by inoculation of the strain *Amorphotheca resinae* ZN1 (Chinese General Microorganisms Collection Center, Beijing, China; registration number: CGMCC 7452) to remove specific inhibitor compounds. Afterwards, the biodetoxified corn stover was hydrolyzed with the enzyme Youtell #6 at a loading of 135 FPU g^−1^ of substrate, and fermented by the strain *S. cerevisiae* DQ1(Chinese General Microorganisms Collection Center, Beijing, China; registration number: CGMCC 2528). The SSF consisted of a 12 h of prehydrolysis at 50 °C and pH 4.8, followed by a reduction of temperature at 37 °C to promote the growth of the yeast and its sugars fermentation into ethanol. The results showed that the helical agitation during the pretreatment allows the increment of the sugars released during the hydrolysis, giving 81.9 g L^−1^ of glucose compared to the low value of 55.8 g L^−1^ obtained without mixing. As regards the ethanol production, 56.2 g L^−1^ were obtained after 48 h of fermentation instead of 44.4 g L^−1^ reached when no agitation was employed during the pretreatment.

#### Production of ethanol from SSF/CBP in fed-batch/continuous operation mode

Very few works about the simultaneous saccharification and fermentation (SSF) of corn stover in a continuous stirred tank reactor (CSTR) are available (Wooley et al. 1999; Jin et al. 2013), due to the limits related to the experimental troubles, although this system increases the volumetric productivity, mostly when more tank reactors are used (Brethauer and Wyman 2010). Brethauer et al. (2014) performed also a continuous simultaneous saccharification and fermentation (cSSF) of acid-pretreated corn stover using a 3-stage CSTR system. Hydrolysis and fermentation were performed in a 3-L fermentor as described earlier. They observed that at the same ethanol yield of 70 %, an ethanol productivity of 0.24 g L^−1^h^−1^ was observed in the 3-stage continuous system. Moreover, in 8 h of residence time, the single cSSF vessel reached the maximum ethanol productivity of 0.4 g L^−1^h^−1^, a value that dropped when the total resident time was kept constant and the number of vessels was increased. In other experiment, when the total residence time was 24 h, the productivity increased from 0.25 to 0.28 g L^−1^h^−1^ when changed from one to three vessels, respectively.

Jin et al. (2013) evaluated the conversion of AFEX pretreated corn stover into ethanol also by continuous SSCF using a CSTR equipped with five bioreactors connected in series. The first reactor was used for the enzymatic hydrolysis at 50 °C, pH 4.8, and 800 rpm; then, the 24-h prehydrolysate was pumped in the next reactor for the SSCF at 30 °C, pH 5.5, and 220 rpm. This system reached the highest volumetric productivity of 0.46 g L^−1^h^−1^, value 2.3 and 1.8 higher than that observed in the batch SHF and SSCF, respectively. The use of high solids content of lignocellulose biomasses could decrease the loss of sugars, waste of water and steam generation, and also to lead an increment in the rate of bioconversion into ethanol (Kristensen et al. 2009). In spite of this, the high solids loading is related to operative issues as the high viscosity and the little free water, that limit the pretreatment method which can be performed (Modenbach and Nokes 2012).

### Bioreactors for wheat straw conversion

#### Production of sugars from SHF

Several studies regarding the design of the bioreactor systems to be employed in the saccharification of the wheat straw at high solids loading and, that ensuring an effective mixing and a high bioconversion yield, were so far reported (Jørgensen et al. 2007a; Szijártó et al. 2011a, b). Ludwig et al. (2014) designed a new vertical stirred tank reactor supplied with a **segmented helical stirrer** (Fig. 3f) to hydrolyze the wheat straw, after alkaline-pretreatment, at high solids loading. Before testing the new system, they carried out a central composite response surface analysis to optimize the hydrolysis conditions, minimizing the enzyme dosage and maximizing the fiber concentration. The optimization was carried out in 250-mL Erlenmeyer flasks at 50 °C by using the Cellulase Cellic® CTec2 (Novozymes A/S) as hydrolytic enzyme. After 48 h, a glucose yield of 70 % was reached at the optimal solid concentration of 20 % (*w*/*w*) and an enzyme dosage in the range of 20–30 FPU g^−1^ dry matter. When the hydrolysis was performed in the new stirred reactor, at the same optimized conditions and at a speed of 80 rpm, the glucose yield was increased from 70 to 76 %, corresponding to a hydrolysate with 110 g glucose kg^−1^ biomass. The swelling of the fibers generated by the high hemicellulose content of the wheat straw did not allow exceeding the 20 % (*w*/*w*) of dry matter. Riedlberger and Weuster-Botz (2012) reported an accurate high-throughput system suitable for acid or alkaline-pretreated wheat straw, in order to reduce the costs for the optimization of the enzymatic hydrolysis step related to the large use of the enzymes required. The new system, consisting of 48 parallel stirred-tank bioreactors at volume of 10 mL, was equipped with the novel **S-shaped impellers** (Riedlberger and Weuster-Botz 2010) (Fig. 3g). The homogenization of fibers at high solids loading was achieved by two permanent magnets (IBS Magnet, Berlin, Germany) that drove the rotation of the impeller around a fixed axis. Three different solid contents, 4, 8, and 10 % (*w*/*w*) of pretreated dried wheat straw were saccharified with 15 mg protein g^−1^ dry matter and 1.9 mg protein g^−1^ dry matter of Celluclast® and Novozym® 188 (Novozymes A/S), respectively. After 9-h of hydrolysis, the glucose released (∼111 mg g^−1^ dry matter) using the high-throughput system was comparable to the 1-L scale. This test demonstrated the efficient and easy scale-up of the novel system that can be used for the optimization of pretreatment conditions. Other ways to reduce the costs of the saccharification process can be recycling the enzymes or the solid residues, exploiting the ability of adsorption onto lignin and cellulose fractions (Rodrigues et al. 2012; Lee et al. 1994). Pihlajaniemi et al. (2014) investigated the solids-recycling for the hydrolysis of the autohydrolysed wheat straw; moreover, they compared the hydrolysis yield and the volumetric sugars productivity obtained through the solids-recycling for the sequential and the batch reactions, at similar loading of enzymes, substrate, and total liquid. A commercial enzyme mix consisting of cellulase (Econase CE, AB Enzymes), β-glucosidase (Novozyme 188), and xylanase (GC 140, Genencor) was used for the hydrolysis. The reaction used at solids loading of 16 % (*w*/*w*) was carried out in a 5-L reactor composed by a horizontal cylinder and a system of **rotating paddle** (Fig. 3h), at speed rotation of 2 rpm. The hydrolysis yield was almost comparable among the three systems used, whereas differences were observed in the volumetric productivity. Hydrolysis yields of 56 and 59 % were reached in the batch process at 48 and 72 h, respectively. Similar values of 53 % after 48 h and 63 % after 72 h of reaction were obtained for both sequential hydrolysis and solids-recycling processes. Regarding the productivity, 1.4 and 0.8 g L^−1^ h^−1^ were obtained in 48 and 72 h of batch hydrolysis, respectively, using enzyme dose of 9 FPU g^−1^. Values of 54 and 30 % higher were observed when the hydrolysis was performed in sequential hydrolysis or solids-recycling systems, at 48 and 72 h, respectively. Although the hydrolysis yields were comparable and the productivity values were slightly lower at 72 h than at 48 h of the processes, both the solids-recycling and sequential hydrolysis system could be applied for an efficient enzymatic hydrolysis of the pretreated wheat straw.

#### Production of ethanol from SSF/CBP in batch operation mode

The use of lignocellulosic biomasses at solids loading above 15 % (*w*/*w*) of dry matter is required in order to obtain an ethanol concentration more than 4 % (*w*/*w*) and thus, making economically feasible the bioconversion process (Fan et al. 2003; Wingren et al. 2003). The high solids loading needs a mixing system to reduce the problematic related to the liquefaction and saccharification steps, like the initial viscosity and the high concentration of the inhibitory compounds. Jørgensen et al. (2007b) designed a reactor system useful for an efficient mixing during the liquefaction and saccharification of the pretreated wheat straw at low speed rates. A reactor consisted of a horizontally placed drum divided into five independently sections equipped by **three paddlers** (Fig. 3i) that were assembled around a horizontal rotating shaft. They investigated the effects over the liquefaction and glucose releasing varying the mixing speed and the initial dry matter content, carrying a liquefaction and saccharification steps for 96-h treatment; moreover, they evaluated the yield of ethanol after 8-h liquefaction and presaccharification followed by 84 h SSF. The enzyme cocktail consisting of Celluclast 1,5 FG L and Novozym 188 (Novozymes, Denmark) was used for the saccharification The mixing speed range tested was between 3.3 and 11.5 rpm, while the dry matter content went from 20 to 40 %. They observed that, after 24 h of treatment, the wheat straw structure was completely liquefied at low speed of 3.3 rpm. They also reported that the mixing speed did not influence the cellulose conversion in the tested range; differently, the hemicellulose conversion was influenced in a negative way, since it decreased 18 % when the mixing speed increased from 3.3 to 11.5 rpm. Regarding the effect of the dry matter, they obtained a maximum of 86 g glucose kg^−1^ of biomass after 96 h of treatment at solid loading of 40 % (*w*/*w*). As reported in other works (Ingesson et al. 2001; Lu et al. 2002; Tengborg et al. 2001), although the maximum glucose released was reached at the highest initial dry matter, a decrement of the bioconversion yield based on the total initial cellulose content, was observed when the dry matter was increased. When they evaluated the effect of different initial dry matter (from 2 to 40 % (*w*/*w*) over the 84 SSF process, after 8 h of liquefaction and pre-saccharification at 50 °C, the highest ethanol concentration of 48 g kg^−1^ of biomass was obtained at 35 % (*w*/*w*) dry matter after 144 h. According to other experiments (Mohagheghi et al. 1992; Devantier et al. 2005), a drop of the fermentation performance at value of dry matter higher than 35 % (*w*/*w*) was observed, due to the stress conditions (i.e., high osmotic pressure, ethanol, and inhibitor concentration) determining the loss of viability of the yeast.

#### Production of ethanol from SSF/CBP in fed-batch operation mode

In order to obtain a high yield of ethanol from the bioconversion of the lignocellulosic biomasses, it is necessary to convert all the available sugars, hexoses and pentoses, that are both present in the macromolecular structure. Olofsson et al. (2010a) reported how the SSCF process of the acid-pretreated wheat straw, combining the fed-batch and the enzyme feeding, improves the glucose and xylose co-fermentation of the recombinant xylose-fermenting strain *S. cerevisiae* TMB3400 (Wahlbom et al. 2003). Through the process, the glucose was released at a very low rate, improving the xylose uptake by the yeast (Olofsson et al. 2008, 2010b). A 2.5-L bioreactors (Biostat A. B. Braun Biotech International, Melsungen, Germany; Biostat A plus; Sartorius, Melsungen) was used for the process in anaerobic conditions (Palmqvist et al. 1996). The saccharification of wheat straw was performed by using the enzyme mix consisting of the Xylanase XL (SAF-ISIS, Souston, France) and Novozyme 188 (Novozymes, Denmark). In all the SSCF experiments, the feed of the substrate was performed after 6, 12, 18, and 24 h, starting from the solids loading of 8 % until reaching the value of 11 %. Regarding the enzymes feed, four different profiles, namely A, B, C, and D, were tested; in all cases, a low initial amount of enzyme was added to improve the liquefaction of the substrate. In the profiles A and B, the enzymes were added until 24 and 48 h, respectively; instead, in the profiles C and D, the enzymes were added for the first time during the last addition of the substrate and carried out until 48 h. The profile D differs from C since at 24 h an additional feed of yeast was made, in order to evaluate if a high yield of ethanol could be achieved. As reference experiment, a SSCF in which the feeding of substrates was carried out as described earlier, while the total amount of the enzymes was added at the beginning of the process, was performed. In comparison with the reference experiment, while the other profiles did not gave improvements, profile B gave the best results, allowing an increment from 40 to 50 % of the xylose conversion, from 0.31 to 0.35 g g^−1^ of ethanol yield and from 33 to 38 g L^−1^ of the final ethanol concentration.

### Bioreactors for other biomasses conversion

#### Production of sugars and ethanol from SHF in batch operation mode

Gupta et al. (2012) exploited the saccharification and ethanol production of pretreated mesquite wood in batch experiment. Enzymatic hydrolysis of sodium chlorite-pretreated lignocellulosic biomass was performed in a 3.0-L stirred tank reactor (STR) equipped with Rushton impeller (Fig. 3d) for shaking (150 rpm), heating jacket and heat exchangers for temperature control (50 °C), using 22 FPU g^−1^ of dry substrate (gds) of cellulase (Sigma-Aldrich) and 68 U β-glucosidase/gds (Sigma-Aldrich). Fermentation was performed at 30 °C, a constant speed of 200 rpm, and an aeration of 0.4 vvm, inoculating a *S. cerevisiae* strain to the hydrolyzed slurry after the addition of nutrients (3 g L^−1^ yeast extract, 0.25 g L^−1^ ammonium phosphate dibasic) with an initial pH 6.0. Enzymatic hydrolysis, performed using four different substrate loading values (5, 10, 15, 20 % *w*/*v*) showed that a significant increment in sugars concentration was observed at increasing biomass concentration up to 15 % (from 41.10 to 90.07 g L^−1^) declining thereafter at the highest substrate level (80.78 g L^−1^ with 40.39 % cellulose conversion). After 11 h of fermentation, a concentration of 34.78 ± 1.10 g L^−1^ (corresponding to a yield of 0.45 g g^−1^ and a productivity of 3.16 g L^−1^ h^−1^) was reached.

Innovative bioreactors were also developed to use wastes rich in lignocellulosics and residues from industrial and agricultural processes for bioethanol production. Caspeta et al. (2014) developed a system of six units of 30-mL mini-bioreactor (nominal volume) with a **peg-mixer** (Fig. 3l) and a jacked-glass vessel with olives used for water circulating and temperature control during enzymatic hydrolysis, at 50 °C, and sugar fermentations at 37 °C, to improve the SHF process of agave bagasse. Hydrolysis has been conducted using 15 FPU of Celluclast 1.5 L (NS50013, Novozymes) and 30 CBU of Novozyme 188 (NS50010, Novozymes) per gram of solids, at speed of 150 rpm by a compact overhead stirrer. Using high solids loading (20 %, *w*/*w*), this system enhanced saccharification giving 120 g L^−1^ of glucose, corresponding to 80 % of cellulose conversion after 24 h and a maximum glucose yield of 135 g L^−1^, corresponding to 90 % cellulose conversion after 64 h. Ethanol production reached values of 64 L^−1^ after 9 h of culture with *S. cerevisiae* strain SuperStart.

Direct conversion of fruit and citrus peel wastes (CPW) into bioethanol, without pretreatment, was investigated by Choi et al. (2015). Hydrolysis of CPW was performed using 12–16 and 10–25 mg protein g^−1^ fruit waste of two enzymes produced in-house from *Aspergillus citrisporus* (Korean Culture Center of Microorganisms KCCM6507) and *Trichoderma longibrachiatum* (Korean Collection for Type Cultures KCTC 6507), in citrate phosphate buffer (pH 4.8) at 45 °C for 48 h at a speed of 180 rpm. Fermentation was conducted in continuous mode at 30 °C in 80-mL immobilized cell reactor (ICR) where *S. cerevisiae* cells was immobilized in alginate drops. A d-limonene removal column (LRC), containing raw cotton and activated carbon, was also joined to the fermentation reactor for desorption of potentially inhibitor substances from the hydrolyzate. About 90 % of CPW enzymatic conversion into fermentable sugars was reported after 48 h. The CPW hydrolyzed was fed into the reactor from the feed stock by peristaltic pump at a flow of 0.08 mL min^−1^. Fermentation in the LCR-ICR system resulted in high ethanol concentrations reaching values from 14.4 to 29.5 g L^−1^ (ethanol yields 90.2–93.1 %) that were 12-fold higher than ethanol values recovered in the ICR fermentation performed without LCR.

Okur and Saraçoglu (2006) reported that the aeration rate largely effected the ethanol formation from hydrolyzed crop residues in a bioreactor in uncontrolled pH conditions. Acid hydrolysis of sunflower seed hull, performed using a relatively low temperature (90 °C) and low H_2_SO_4_ concentration (0.7 M), allowed recovering approximately 90 % of sugars from hemicellulose. Detoxified acid hydrolyzed, containing 35–40 g L^−1^ of total reducing sugars, was used for ethanol production with the yeast *Pichia stipitis*. Fermentation was performed in a batch culture bioreactor system consisting of a 0.6-L glass flask with a Teflon and silicone-lined top cap, a Teflon and glass impeller on the top cover, and a paddle blade magnetic impeller (Fig. 3c) on the bottom of flask. The fermentation process was carried out at 30 °C with agitation of 100 rpm and the air was sparged by a flowmeter from the bottom of the vessel at different aeration rates (0, 2.88, 5.76, 7.99 vv^−1^ min^−1^). Authors reported that oxygen supply stimulated yeast growth and ethanol formation although depending on aeration rate. In fact, the highest sugar consumption (78 %), ethanol concentration (9.66 g L^−1^) and ethanol yield (0.41 g g^−1^) was reported at the lowest tested flow rate (2.88 vv^−1^ min^−1^).

#### Production of sugars and ethanol from SHF in fed-batch operation mode

SHF fed-batch experiments were performed by Gupta et al. (2012) using pretreated Mesquite wood in comparison with the batch system described earlier. Enzymatic hydrolysis was carried out in a 3.0-L STR as described above with an initial solids concentration of 5 % and adding 11 FPU/gds of cellulose, 34 U β-glucosidase/gds, and 5 % of solids after 24, 56, and 80 h. The use of a fed-batch system resulted in a further increase of sugars production (127 g L^−1^ with 63.56 % cellulose conversion) of 56 % respect to the batch system with a WIS content of 20 %. The highest sugars content resulted also in the highest ethanol concentration in fed-batch process. In fact, ethanol production of 52.83 g L^−1^ (ethanol yield and productivity of 0.45 g g^−1^ and of 4.40 g L^−1^ h^−1^, respectively) was observed.

#### Production of ethanol from SSF/CBP in batch operation mode

Switchgrass (*Panicum virgatum* L.) is adopted as a model energy crop by the US Department of Energy due to its high biomass yield, grown in different climate conditions and suitability for marginal land use (Kim et al. 2015); therefore different studies have focused on bioethanol production from this crop. Isci et al. (2009) performed SSF process of ammonia-soaked switchgrass using a 50-L (working volume) pilot-scale bioreactor. Pretreated switchgrass biomass soaked in ammonium hydroxide (containing 48 % cellulose, 23 % hemicellulose, and 22 % Klason Lignin) has been directly used for SSF experiments in a 50-L steam-jacketed fermenter equipped with three Rushton-type impellers (Biostat U-50, Sartorius) (Fig. 3d). SSF was conducted at 35 °C and 130 rpm for 72 h after aseptical addition of yeast inoculum and 77 FPU g^−1^ cellulose of cellulase enzyme (Spezyme CP, Genencor Int.). At the end of the process, authors observed an ethanol yield of 73 %.

Rotary drum reactor represents another interesting strategy to improve the homogenization of pretreated lignocellulosic biomass in SSF. Lin and Lee (2011) used this technology to optimize the SSF process of alkaline-pretreated cogon grass. Pretreated biomass was loaded at a quantity of 1 kg (10 % WIS concentration, *w*/*w*) in a 5-L rotary drum reactor and SSF process has been run using 0.258 mL g^−1^ WIS of enzyme Accellerase 1500 and Ethanol Red dry *S. cerevisiae* yeast (1 g L^−1^ dry yeast) at 37 °C and initial pH of 5.0. The reactor was rotated at 5 rpm for 1 min at 0, 24, 48, and 72 h. An ethanol concentration of 19.1 g L^−1^ has been obtained corresponding to 76.2 % of the theoretical ethanol yield.

These results were confirmed scaling up SSF process in a 100-L rotary drum reactor using alkaline-pretreated sugarcane bagasse (Lin et al. 2013). The reactor was arranged by a double-cone rotary reactor providing a double-wall structure for controlling temperature by circulating either cold or hot water between the double walls. Moreover, it was equipped with wave-shaped baffles along the inner wall surface of the vessel and with CO_2_ outlet port. In this reactor, 10 kg of alkaline-pretreated sugarcane bagasse (WIS concentration of 10 %, *w*/*w*) has been processed by SSF at 42 °C for 72 h by using a commercial cellulase Accellerase 1000 (0.2 mL g^−1^ WIS) and *Kluyveromyces marxianus* var. *marxianus* (0.5 g L^−1^). The reactor was rotated at 5 rpm for 1 min only at the beginning of the process and every 24 h. After 72 h, 24.6 g L^−1^ of ethanol concentration (79.0 % ethanol yield) were obtained.

A particular reactor system has been set up by Ishola et al. (2013) using simultaneous saccharification, filtration and fermentation (SSFF) process. This system included three integrated units: hydrolysis and fermentation vessels among which fermentation broth was circulated by filtration system. In particular, pretreated spruce chips (SO_2_-catalized steam explosion) with 10 % suspended solids (SS) was mixed with 35 FPU g^−1^ SS of the commercial enzyme Cellic® CTec3 (Novozymes) in a 2.5-L reactor (Infors AG107504, Minifors, Switzerland) and pre-hydrolyzed for 24 h at 50 °C, pH 5.0, and agitation of 500 rpm. During SSFF process, hydrolyzed slurry was continuously pumped at flow of 0.8 L min^−1^ in the fermentation vessel (1.5-L bioreactor, Biostat®B plus 8843414 Sartorius, Germany) by a cross-flow membrane and simultaneously, the flow of the liquid of fermentation vessel was inverted to the hydrolysis reactor. Moreover, with the aim to equilibrate the uptake in the fermentation reactor, another peristaltic pump pushed the permeate out of the filter module by increasing flow rate from 1.1 to 2.9 mL min^−1^. To ensure yeast culture sedimenting, the fermentation bioreactor was equipped with a settler. The SSFF process was conducted for 96 h and 31.1 ± 1.2 g L^−1^ ethanol (theoretical yield of 85.0 %) were reached.

Svetlitchnyi et al. (2013) used consolidated bioprocessing (CBP) approach for producing ethanol from poplar wood through thermophilic bacteria without the need for additional cellulolytic enzymes. In particular, washed and unwashed solid fraction of poplar wood obtained after dilute sulfurous acid steam explosion were loaded in 2-L stirred vessel fermentor (Biostat B-DCU, B. Braun/Sartorius AG) equipped with double jackets for temperature control, two Rushton type stirrer blades (Fig. 3d), pH control loops and high-precision blow-off valves for pressure controlling in a range of 1.3–1.5 bar. CBP process was conducted at a constant pH of 6.75 and temperature of 72 °C, inoculating the cellulolytic/xylanolytic strain *Caldicellulosiruptor* sp. DIB 004C (GenBank accession number JX988415) and the fermenting thermophilic ethanologenic/xylanolytic strain *Thermoanaerobacter* DIB 097X (GenBank accession number JX988424) in monocultures and in dual co-cultures. Authors reported that the CBP approach with operating temperatures above 70 °C and developing co-cultures of these bacterial strains led to an efficiently conversion of C6- and C5-sugars from pretreated lignocellulosic material into ethanol (up to 34.8 mM) and other products (33.6 mM) such as lactate and acetate.

#### Production of ethanol from SSF in fed-batch operation mode

Isci et al. (2009) scaled up SSF process of ammonia-soaked switchgrass using a 350-L steam-jacketed fermenter equipped with three-blade axial flow impeller (Model PTT, Walker Stainless Equipment Co.). The process was carried out at 200 rpm for 120 h in semiaseptic and fed-batch conditions, adding pretreated ammonia-soaked switchgrass biomass at three times (0, 5, and 24 h) to allow the thinning of substrate by cellulase (77 FPU g^−1^ cellulose). Controlling bacterial contamination during the process and improving stirring conditions of pretreated lignocellulosic biomass, it is possible to enhance bioethanol production ensuring the success of SSF scale-up. In fact, authors reported that in the best case in 350-L fermenter the ethanol yield was 74 %, similar to that obtained in 50-L fermenter (ethanol yield 73 %).

Han et al. (2014) developed SSF reactor to generate high-concentration bioethanol from *Miscanthus* biomass. They used a continuous twin-screw extruder for pretreating lignocellulosic biomass. The pretreatment reactor was fed with *Miscanthus* biomass at a rate of 18 g min^−1^ and pretreatment solution at 90 mL min^−1^. During this process, performed using a deficient amount of NaOH at 95 °C with a rotation of 80 rpm, solid and liquid components were separated using an oil press to reuse the solution obtained after pretreatment. This approach allowed reducing the costs of pretreatment process minimizing wastewater and reducing the amount of expensive alkali catalysts used. The resulting pretreated biomass was continuously fed at a rate of 80–150 g h^−1^ into the bottom of a 5-L tank reactor for SSF until to achieve a final concentration of approximately 25 % (*w*/*v*), containing a glucose concentration of 40 g L^−1^. SSF process has been conducted at 32 °C with agitation (90 rpm) for 96 h using an enzymatic loading of 30 FPU g^−1^ cellulose (Cellic® CTec2, Novozymes), 15 % Cellic® HTec2 (Novozymes) and 7 % (*v*/*v*) *S. cerevisiae* CHY 1011. The optimized pretreatment process coupled to a fed-batch approach increased the efficiency of hydrolytic enzymes obtaining ethanol at high concentration (up to 74.5 g L^−1^ with a yield and productivity of 89.5 % and 1.4 g L^−1^ h^−1^) using high solid loadings lead to a reduction of distillation energy costs.

Similar approach was also assayed by Kim et al. (2013) that used a continuous twin screw-driven reactor (CTSR) pretreatment associated to a fed-batch SSF for bioethanol production from poplar sawdust. The reactor was composed of 30 segments for continuous biomass rotation, pulverization, and pressure. In this case, diluted H_2_SO_4_ (4 %) was used as catalyst in the pretreatment process conducted at 180 °C with a screw rotation speed of 60 rpm and biomass feeding rate of 1 g min^−1^. Fed-batch SSF experiments were conducted using 30 FPU of Celluclast 1.5 L (Novozymes) and 70 pNPG Novozyme 188 (Novozymes) per gram of cellulose in a bioreactor composed of four units of 1 L in which 6.0 wt % pretreated biomass was added at three stages, maintaining a constant solids concentration. This approach showed a great potential since high ethanol concentration (39.9 g L^−1^) was achieved.

## Conclusions

This review gives an overview of the last advances in the bioreactor configurations used for the conversion of dedicated energy crops and residual materials, describing how parameters like high solids loading, particles size, enzymes recycling, speed/power input, volume, and substrates reactivity, can improve the sugars release and the ethanol concentration. In the last decade, due to the complexity of the lignocellulose macromolecular structure, new bioreactor configurations have been designed and/or applied in order to make feasible the use of high substrate loading during the bioconversion process. Bioreactors equipped with new agitation systems like a special segmented helical stirrer (Ludwig et al. 2014), the S-shaped impellers (Riedlberger and Weuster-Botz 2012) and the double helical ribbon impeller (Du et al. 2014) were constructed to achieve an efficient fiber homogenization, reducing the required energy in conditions of high substrate loading. These agitation configurations enhance the homogeneous mixing of the biomass counteracting the elevate initial viscosity, due to the high biomass dosage, and allowing to profit several advantages related to this condition. As a matter of fact, a high substrate loading can lead to several economic and operative advantages such as the reduction of reactor size, the decrease in the sugars loss and wastes generation and easier downstream processing, due to higher product concentration. However, further developments in the bioreactor configuration combined to new efficient agitation systems and optimal operative conditions are needed to apply the process in pilot or industrial scale and to achieve a high bioconversion yield.
